# Vision-based reconstruction of laser projection with invariant composed of points and circle on 2D reference

**DOI:** 10.1038/s41598-020-68901-7

**Published:** 2020-07-17

**Authors:** Guan Xu, Fang Chen, Rong Chen, Xiaotao Li

**Affiliations:** 1 Transportation College, Nanling Campus, Jilin University, Renmin Str. 5988#, Changchun, China; 2 School of Mechanical and Aerospace Engineering, Nanling Campus, Jilin University, Renmin Str. 5988#, Changchun, China

**Keywords:** Imaging and sensing, Optical metrology

## Abstract

A vision-based reconstruction method is conducted by the point-circle invariant and the planar laser. The planar laser is coplanar to the two-dimensional (2D) reference. The combination of a circle on the 2D reference, a point on the 2D reference and a random point on the laser stripe is considered as the invariant, which is impervious to the projection from the laser plane to the image. Therefore, the reconstruction model is achieved by the invariant, which is generated from the projections of the above geometric features. The experiments are performed to verify the performance and reconstruction error of the method. The minimum error is 0.473 mm for the camera-reference distance of 600 mm, the scaleplate-projector distance of 100 mm and the reference length of 30 mm. Besides, the maximum error is 4.960 mm, for the camera-reference distance of 900 mm, the scaleplate-projector distance of 400 mm and the reference length of 120 mm. The reconstruction error means of 0.891 mm, 1.365 mm, 2.578 mm and 3.767 mm are observed with respect to the test conditions of 600–900 mm, which proves the applicability of the reconstruction method with the point-circle invariant.

## Introduction

The non-touch measurement of the 3D object surface is an attractive problem with potentials in the extensive fields of optical inspection, e.g. product quality inspection^1,2^, face recognition^3,4^, robot^5,6^ and medical science^7,8^, geography^9^.

The camera is a convenient information collection device for the non-touch measurement due to its high resolution and non-touch characteristics^10^. However, the camera only realizes the transform from the spatial surface to the image. Without the prior knowledge, the inverse problem of the above transform for the camera is a typical ill-conditioning problem^11–13^. Therefore, the camera-based 3D surface reconstruction is performed by two cameras or one camera with a laser projector. The two cameras’ method is applicable to the object with the obvious feature points, feature lines, feature structures^14–16^. Hence, even though the additional camera complements the lack of one degree of freedom of the one-camera system, it often fails to achieve the 3D reconstruction of the continuous surface without features. The other method is conducted by the camera accompanied by a laser projector. The essential one degree of freedom for the 3D reconstruction is indicated by the coordinate or phase position information of the laser projector^17,18^. The 3D surface is derived from the assistance of the active light mark from the projector.

Several vision-based methods are developed to perform the test of 3D surface. Sabe et al.^5^ proposes a stereo-vision system to profile the obstacle ahead of a robot. The obstacle inspection and localization results are described in an indoor environment. The Hough transform^19^ is employed for the detection of the floor plane. Faessler et al.^6^ presents a vision-based 3D mapping method based on the quadrotor aerial vehicle. The motion estimation thread consists of image alignment, feature alignment and structure refinement. The 3D thread includes feature extraction, depth filter updating and converging. Li et al.^7^ describes a 3D facial anthropometry of infant lips with the structured light. The anthropometry measurement consists of stereo cameras and a structure light generator. Triangulation is established by the camera, the projector and the measured lip. The color information is also derived from the inspection system. Stančić et al.^8^ outlines a structured light method that complies with the anthropometric parameter requirement. New light pattern is designed to realize the more accurate and robust measurement. Olson et al.^9^ addresses the mapping method of the terrain for the motion robot on Mars. The wide-baseline technology is utilized by moving a single camera to different places, instead of the conventional stereo vision. The motion estimation and robust matching are addressed to overcome the relative position estimation of the robot and the large variation of views. The volumetric measurements are compared to the conventional method results. Guan et al.^20^ provides an approach to profile the 3D surface from the distortions of the structured light pattern. The structure light pattern used is a combination of multiple patterns. Marin et al.^21^ presents a methodology to decide the number of patterns and the calculation of the fringes, which enhances the reconstruction precision from the noise of the structured light system. Among the measurement methods above, the stereo-vision-based methods take the advantage of the non-touch feature for 3D reconstruction. The stereo-vision-based methods require the robust representations of the features on the surface and are unavailable for the continuous surface without features. The structured light methods actively generate and project the coded light onto the measured surface. Therefore, the advantage of the structured light method is the adaptability for both the continuous surface and the discontinuous surface. However, the coded light derived from the LCD or DLP projector is prone to be interfered by the environmental light field. Thus, the laser projector is more robust for the 3D reconstruction in the normal environment.

In the paper, the adopted laser projector generates a laser plane for the active marking. The camera obtains the image of the bended laser stripe on the measured object. As the projectivity from the point on the bended laser stripe to the point on the image plane is a homography, i.e. the point on the laser stripe is different to the related image point. Therefore, the point on the laser stripe cannot be induced by the image point directly. However, there is an invariant in the transform process from the 2D laser plane space to the 2D image space. A reconstruction method is presented with a circle, a planar laser and the points on the same plane. As the laser is 2D and the intersection laser stripe is also on the same laser plane, the homography invariant derived from two points and a circle is established in the measurement system so as to realize the profiling of the laser stripe. Moreover, the reconstruction model is constructed by the invariant with a circle, a planar laser and the points on the same plane. The high-order equations with two unknowns are avoided in the model by the two axis-points. As the circle is insensitive to the rotation angle in the test, the reconstruction model with the circle, the planar laser and the points is stable compared to the point-laser-based methods. The points and the ellipse that is the projection of the circle can be conveniently extracted by the Harris corner detector^22^ and Hough transform^19^. It is an effectual and convenient reconstruction method, as the value consisting of the two points and the circle is invariable before and after the projectivity. The other sections include: “Solution model” interprets the modeling process of the reconstruction method with the invariant of the two points and the circle. The experiments are analyzed and discussed in “Results”, moreover, the measurement errors are verified in different measurement conditions. “Summary” summarizes the method.

## Solution model

**Figure 1:**
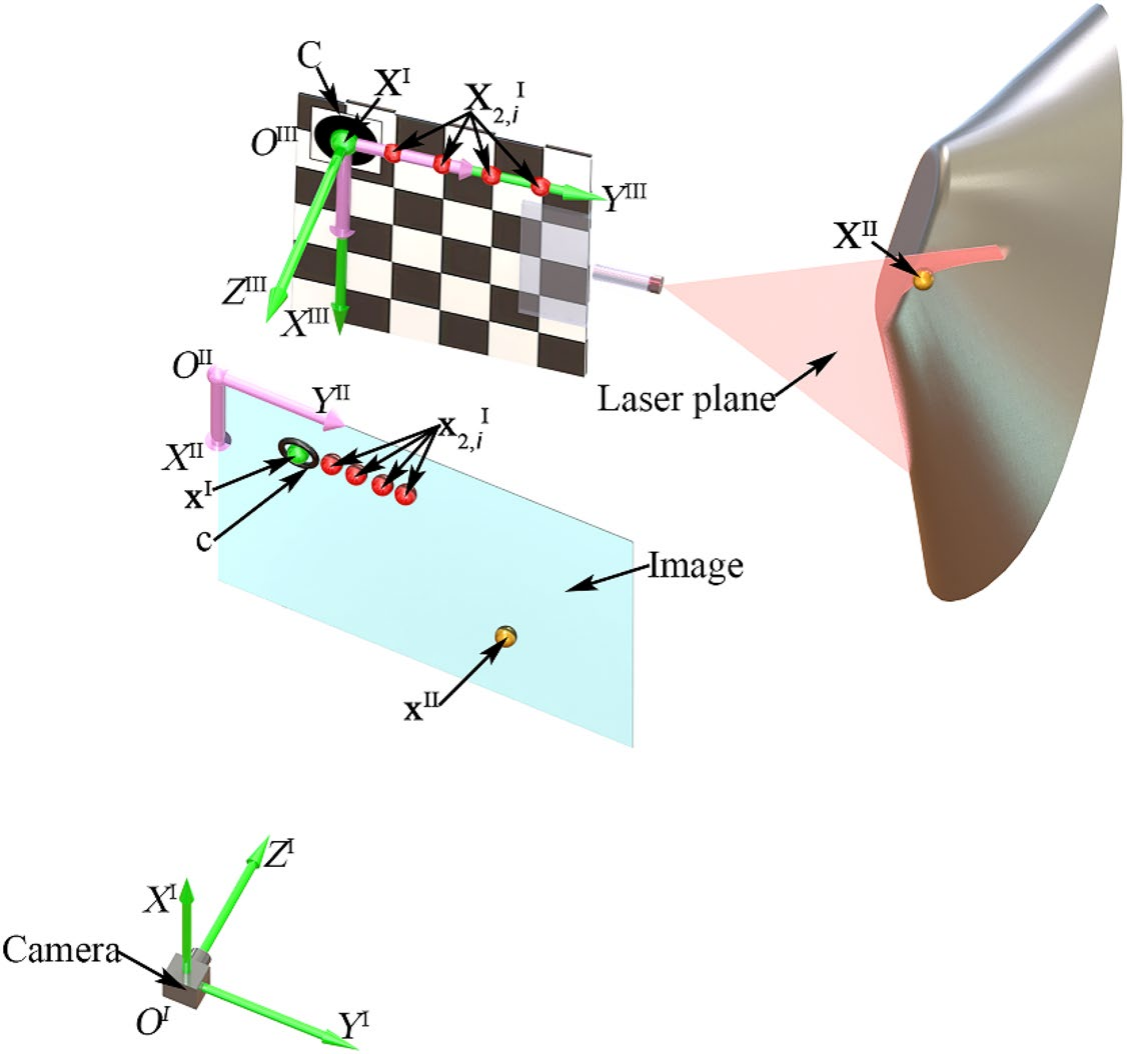
The vision-based model of the reconstruction with the point-circle invariant and the laser plane that is coplanar to the 2D reference.

The vision-based model is described in Fig. 1. The system is comprised of a laser projector, a 2D reference and a camera. A circle with the known radius is printed on the top-left of the 2D reference. The chessboard pattern is adopted to the rest of the reference to provide the feature points for both invariant and calibration. The laser projector is fixed on the front of the reference by the axle and hole coordinating. The laser plane is induced from the projector and in accordance with the same plane of the chessboard pattern. The camera image includes the reference and the intersection light curve on the measured object. The coordinate systems of the reference (CSR), the camera (CSC), and the image (CSI) are indicated by the abbreviations.

The center of the circle on the top-left of the 2D reference is in accordance with the origin of CSR. According to Ref.^23^, the circle on the 2D reference is transformed to the conic on the image by the homography G^24^. Therefore, the transformation is represented by

1 $${\text{S}}_{2} = {\text{G}}^{{\text{T}}} {\text{S}}_{1} {\text{G}}$$

where ${\text{S}}_{2} = [s_{j} ]_{3 \times 3}$ is the image of the circle on the 2D reference in CSI. $${\text{S}}_{1} = \left( {\begin{array}{*{20}c} 1 & 0 & 0 \\ 0 & 1 & 0 \\ 0 & 0 & { - R^{2} } \\ \end{array} } \right)$$ is the origin circle on the 2D reference. R indicates the circle radius on the top-left of the 2D reference in CSR.

The inverse transformation from the image circle to the reference is

2 $${\text{S}}_{1} = {\text{G}}^{{\text{ } - \text{ T}}} {\text{S}}_{2} {\text{G}}^{{ - 1}}$$

According to Ref.^23^, the transformation from the point on the reference to is

3 $${\mathbf{Y}} = {\text{G}}{\mathbf{y}}$$

The inverse transformation of Eq. (3) is

4 $${\mathbf{y}} = {\text{G}}^{ - 1} {\mathbf{Y}}$$

The value consisting of two points and a circle on the plane of the reference is represented by

5 $$E_{1} { = }\frac{{\left[ {({\mathbf{Y}}^{\text{I}} )^{{\text{T}}} {\text{S}}_{1} {\mathbf{Y}}^{{\text{II}}} } \right]^{2} }}{{\left[ {({\mathbf{Y}}^{\text{I}} )^{{\text{T}}} {\text{S}}_{1} {\mathbf{Y}}^{\text{I}} } \right]\left[ {({\mathbf{Y}}^{{\text{II}}} )^{{\text{T}}} {\text{S}}_{1} {\mathbf{Y}}^{{\text{II}}} } \right]}}$$

where *E*_1_ is the value of Eq. (5). ${\mathbf{Y}}^{\text{I}}$ is the point in CSR. ${\mathbf{Y}}^{{\text{II}}}$ is the laser projective point in CSR.

The corresponding value consisting of two points and a circle on the plane of the image is expressed by

6 $$E_{2} { = }\frac{{\left[ {({\mathbf{y}}^{\text{I}} )^{{\text{T}}} {\text{S}}_{2} {\mathbf{y}}^{{\text{II}}} } \right]^{2} }}{{\left[ {({\mathbf{y}}^{\text{I}} )^{{\text{T}}} {\text{S}}_{2} {\mathbf{y}}^{\text{I}} } \right]\left[ {({\mathbf{y}}^{{\text{II}}} )^{{\text{T}}} {\text{S}}_{2} {\mathbf{y}}^{{\text{II}}} } \right]}}$$

where *E*_2_ is the value of Eq. (6). ${\mathbf{y}}^{\text{I}}$ is the image point in CSI. ${\mathbf{y}}^{{\text{II}}}$ is the image laser point in CSI.

Substitute Eqs. (2), (4) into (6), then the property of the values *E*_1_ and *E*_2_ under the projective transformation is

7 $$E_{{1}} = E_{{2}}$$

For the origin point ${\mathbf{Y}}_{1}^{\text{I}} = (0,0,1)^{\text{T}}$ and the laser point ${\mathbf{Y}}^{{\text{II}}} = (X^{{\text{II}}} ,Y^{{\text{II}}} ,1)^{\text{T}}$ in CSR, the invariant value in Eq. (5) is express by

8 $$E_{1,1} = \frac{{ - R^{2} }}{{{(}X^{{\text{II}}} )^{2} + (Y^{{\text{II}}} {)}^{2} - R^{2} }}$$

In the image, the invariant value in Eq. (6) can be derived from

9 $$E_{{2,1}} { = }\frac{{\left[ {({\mathbf{y}}_{1}^{\text{I}} )^{{\text{T}}} {\text{S}}_{{2}} {\mathbf{y}}^{{\text{II}}} } \right]^{2} }}{{\left[ {({\mathbf{y}}_{1}^{\text{I}} )^{{\text{T}}} {\text{S}}_{{2}} {\mathbf{y}}_{1}^{\text{I}} } \right]\left[ {({\mathbf{y}}^{{\text{II}}} )^{{\text{T}}} {\text{S}}_{{2}} {\mathbf{y}}^{{\text{II}}} } \right]}}$$

where ${\mathbf{y}}_{1}^{\text{I}}$, ${\mathbf{y}}^{{\text{II}}}$ are the image projections of the points ${\mathbf{Y}}_{1}^{\text{I}} = (0,0,1)^{\text{T}}$ and ${\mathbf{Y}}^{{\text{II}}} = (X^{{\text{II}}} ,Y^{{\text{II}}} ,1)^{\text{T}}$. S_2_ is the image projection of the circle S_1_ on the 2D reference.

For the point on the *O*-*Y* axis ${\mathbf{Y}}_{i}^{\text{I}} = (0,Y_{i}^{\text{I}} ,1)^{\text{T}} (i > 1)$ and the same laser point ${\mathbf{Y}}^{{\text{II}}} = (X^{{\text{II}}} ,Y^{{\text{II}}} ,1)^{\text{T}}$ in CSR, the invariant value in Eq. (5) is express by

10 $$E_{1,i} = \frac{{(Y_{i}^{\text{I}} Y^{{\text{II}}} - R^{2} {)}^{2} }}{{R^{2} - {(}Y_{i}^{\text{I}} )^{2} }}\frac{{E_{1,1} }}{{R^{2} }}$$

In the image, the invariant value in Eq. (6) can be derived from

11 $$E_{{{2,}i}} { = }\frac{{\left[ {({\mathbf{y}}_{2,i}^{\text{I}} )^{{\text{T}}} {\text{S}}_{{2}} {\mathbf{y}}^{{\text{II}}} } \right]^{2} }}{{\left[ {({\mathbf{y}}_{2,i}^{\text{I}} )^{{\text{T}}} {\text{S}}_{{2}} {\mathbf{y}}_{2,i}^{\text{I}} } \right]\left[ {({\mathbf{y}}^{{\text{II}}} )^{{\text{T}}} {\text{S}}_{{2}} {\mathbf{y}}^{{\text{II}}} } \right]}}$$

where ${\mathbf{y}}_{2,i}^{\text{I}}$ is the image projection of the point ${\mathbf{Y}}_{i}^{\text{I}} = (0,Y_{i}^{\text{I}} ,1)^{\text{T}}$.

Stacking Eqs. (7), (10), (11), the *O*–*Y* coordinate of the laser point is

12 $$Y^{{\text{II}}} = (1/n)\sum\limits_{i = 1}^{n} {(Y_{1,i}^{\text{I}} )^{ - 1} \{ RE_{1,i} [R^{2} - {(}Y_{1,i}^{\text{I}} )^{2} ]^{1/2} (E_{1,1} )^{ - 1/2} + R^{2} \} }$$

Stacking Eqs. (7–9), (12) the *O*–*X* coordinate of the laser point is

13 $$X^{{\text{II}}} = \{ - R^{2} (E_{1,1} )^{ - 1} + R^{2} - \{ (1/n)\sum\limits_{i = 1}^{n} {(Y_{1,i}^{\text{I}} )^{ - 1} \{ RE_{1,i} [R^{2} - {(}Y_{1,i}^{\text{I}} )^{2} ]^{1/2} (E_{1,1} )^{ - 1/2} + R^{2} \} } \}^{2} \}^{1/2}$$

Therefore, the laser point **Y**^II^ can be generated from Eqs. (12) and (13). The laser point is further characterized in CSC, the laser point is finally transformed by^23^

14 $${\mathbf{Y}}^{{{\text{II}} ,{\text{C}}}} = {\text{G}}_{{\text{C}}} {\mathbf{Y}}^{{\text{II}}}$$

where ${\mathbf{Y}}^{{{\text{II}} ,{\text{C}}}}$ is the laser point in CSC. ${\text{G}}_{{\text{C}}}$ is the transformation from CSR to CSC.

## Results

The experiments are performed by the instruments including a 2D reference, a laser-plane projector, a camera with 1,280 × 960 resolution, a scaleplate, a computer, and reconstructed objects, in order to verify the vision-based reconstruction method with the invariant composed of the points and the circle. The 2D reference is covered with a paper printed by the black circle and the checkerboard pattern. The side length of the square on the checkerboard is 60 mm. The radius of the black circle is 55 mm. The wavelength of the laser projector is 650 nm and the power is 5 mW. The laser plane is adjusted to the same plane of the one of the 2D reference.

**Figure 2:**
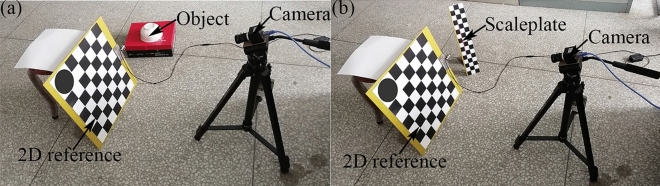
The experiments of the object reconstruction and the system verification by the scaleplate. (**a**) Reconstruction experiment, (**b**) verification experiment.

**Figure 3:**
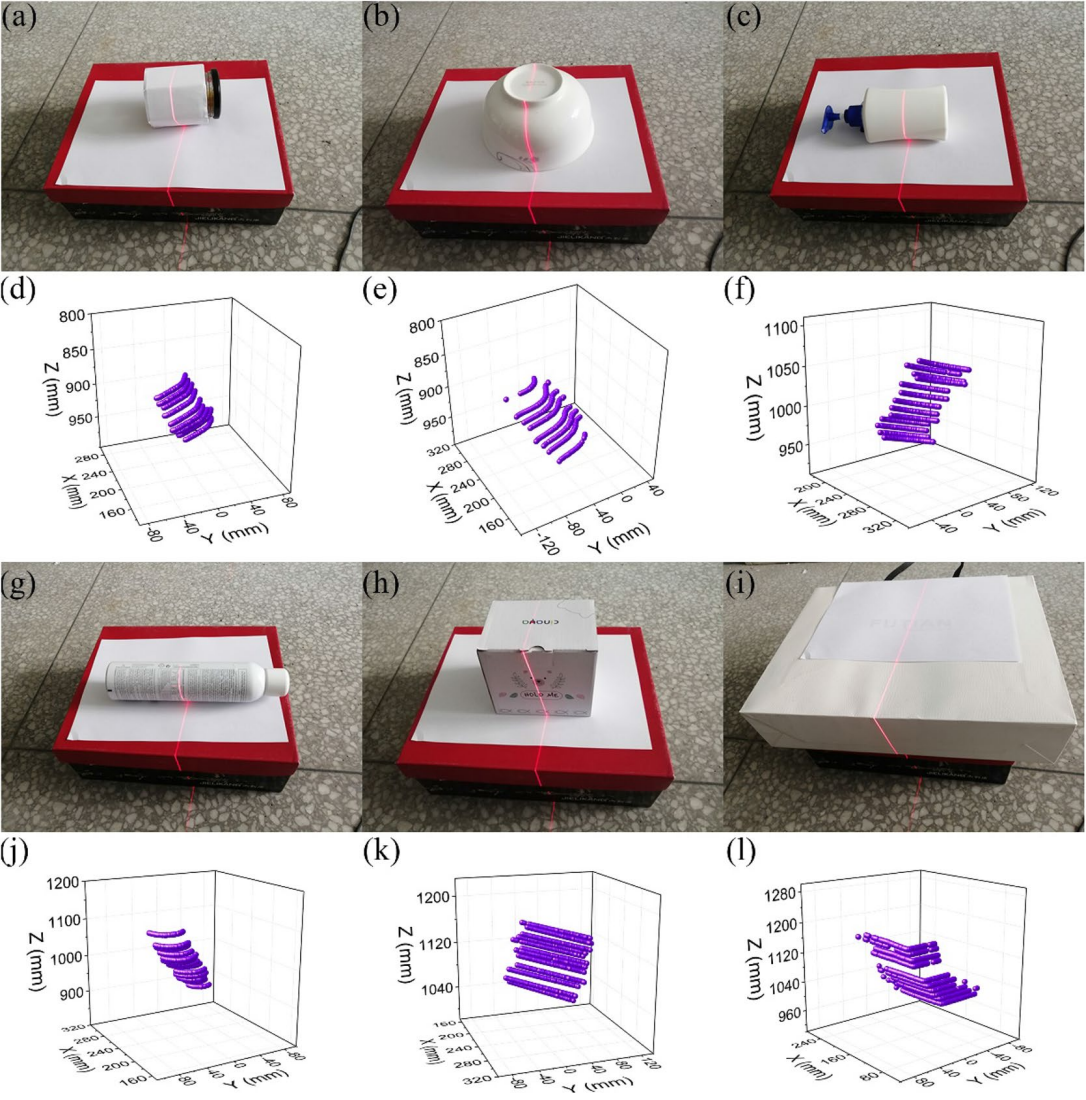
The results of the vision-based reconstruction with the point-circle invariant and the laser plane that is coplanar to the 2D reference. (**a**) A can, (**b**) a bowl, (**c**) a detergent bottle, (**d**–**f**) the reconstruction results of (**a**–**c**), (**g**) a spray bottle, (**h**) a cubic box, (**i**) a case, (**j**–**l**) the reconstruction results of (**g**–**i**).

**Figure 4:**
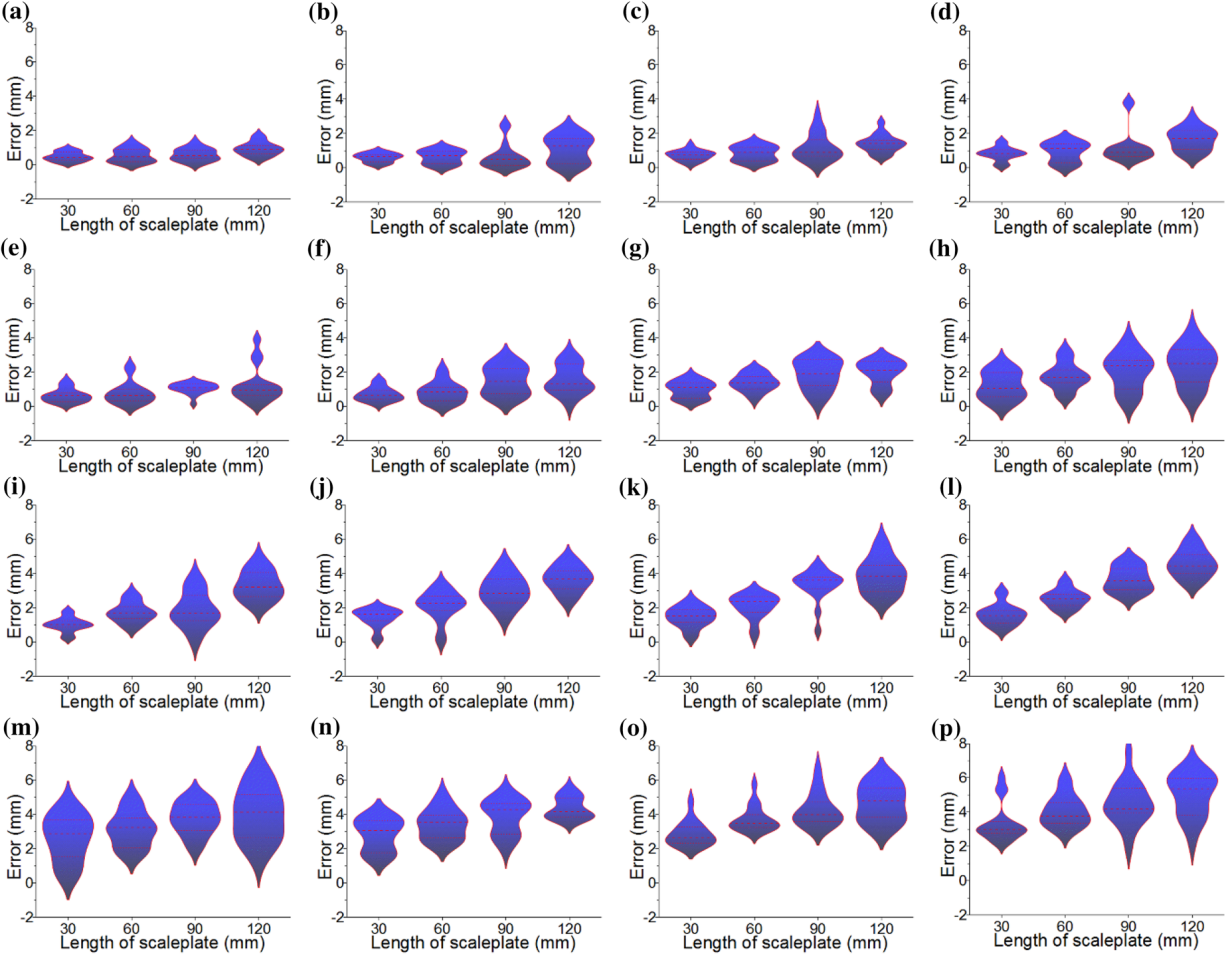
Reconstruction error estimation of the vision-based reconstruction with the point-circle invariant and the laser plane that is coplanar to the 2D reference. (**a**–**d**) the camera-reference distance is 600 mm, the scaleplate-projector distances are 100–400 mm with the 100 mm interval. (**e**–**h**) the camera-reference distance is 700 mm, the scaleplate-projector distances are the same as (**a**–**d**). (**i**–**l**) the camera-reference distance is 800 mm, the scaleplate-projector distances are the same as (**a**–**d**). (**m**–**p**) the camera-reference distance is 900 mm, the scaleplate-projector distances are the same as (**a**–**d**).

**Figure 5:**
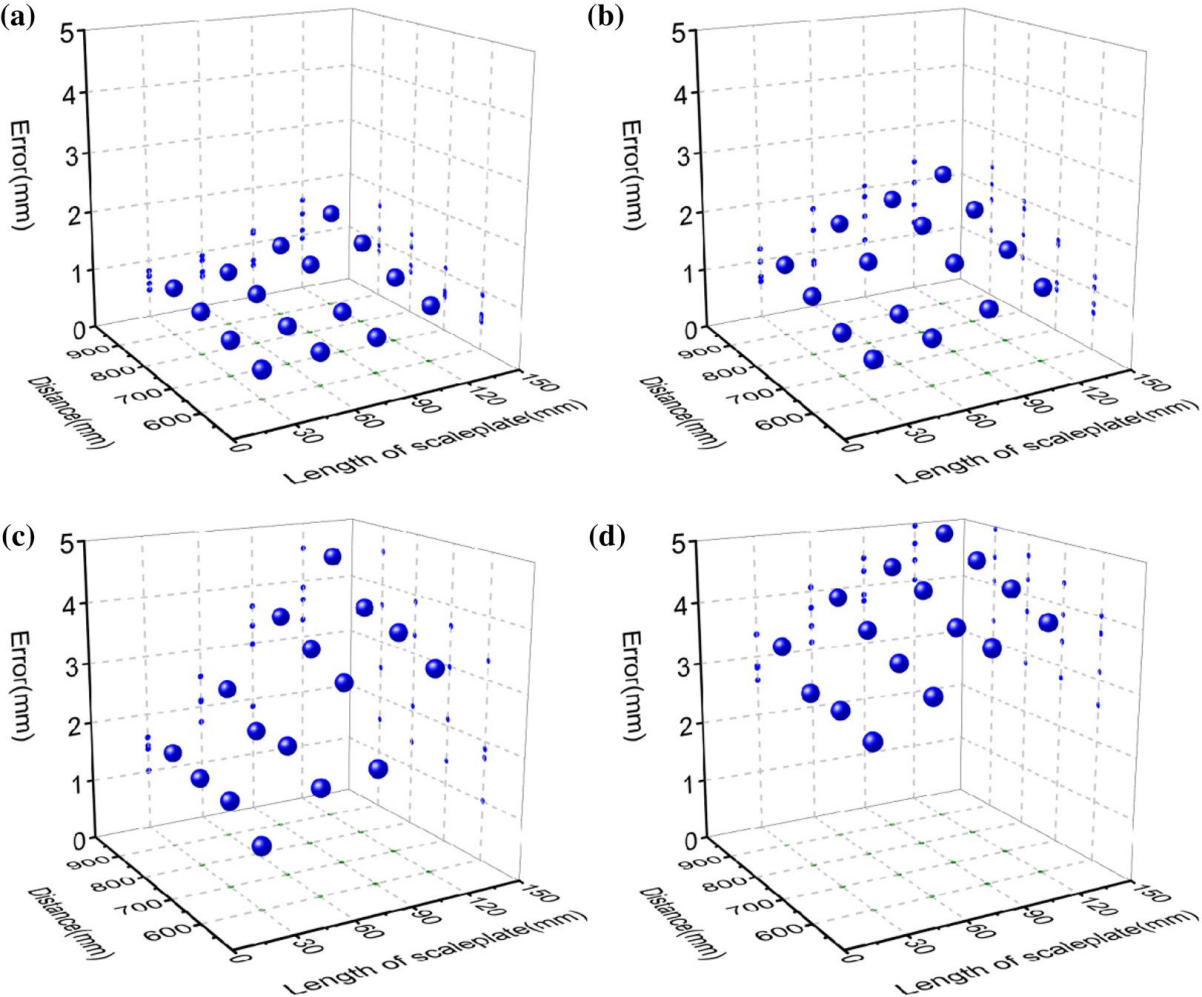
The reconstruction error mean of the vision-based reconstruction with the point-circle invariant and the laser plane that is coplanar to the 2D reference. (**a**–**d**) the camera-reference distances are 600–900 mm with the interval of 100 mm, respectively.

Figure 2a shows the experimental instruments for the reconstruction test. Figure 2b shows the experimental instruments for the reconstruction error test in which a scaleplate is used as the length benchmark. In the reconstruction test, six experimental objects are reconstructed and the reconstruction results are described in Fig. 3. Figure 3a–c,g–i are the physical images of the test objects and Fig. 3d–f,j–1 are reconstructed object contours. Figures 3d–f,j–l show that the reconstructions well meet the actual object shapes in Fig. 3a–c,g–i. The five feature points on the scaleplate are selected and the distances between the two feature points, 30 mm, 60 mm, 90 mm, and 120 mm, are considered as the benchmark lengths. The five feature points are reconstructed under CSC. The error of the reconstruction method is verified by the distances of the recovered feature points and the real point on the scaleplate. Moreover, the error is evaluated with different camera-reference distances and different scaleplate-projector distances. The reconstruction length of feature points and the reference length are used to describe the reconstruction error quantitatively. The absolute values of the reconstruction errors of the vision-based reconstruction with the invariant composed of the points and the circle are indicated in Fig. 4. Figure 5 and Table 1 summarize the averages of the error absolutes in the verification experiments.

**Table 1:** Reconstruction error statistics of the vision-based reconstruction with the point-circle invariant and the laser plane that is coplanar to the 2D reference.

Group (mm)	Distance (mm)	Length of scaleplate (mm)			
		30	60	90	120
600	100	0.473	0.550	0.593	0.931
	200	0.589	0.626	0.678	1.086
	300	0.719	0.840	1.178	1.394
	400	0.811	0.920	1.224	1.648
700	100	0.631	0.772	1.053	1.231
	200	0.707	0.827	1.495	1.560
	300	0.979	1.388	1.850	1.979
	400	1.207	1.766	2.041	2.354
800	100	1.001	1.730	1.872	3.310
	200	1.340	2.111	2.986	3.669
	300	1.459	2.157	3.311	3.880
	400	1.599	2.537	3.650	4.571
900	100	2.595	3.130	3.740	4.017
	200	2.809	3.412	3.852	4.364
	300	2.839	3.724	4.255	4.654
	400	3.372	4.068	4.481	4.960

The camera-reference distance is 600 mm firstly. The distances between the scaleplate and the laser projector on the 2D reference are 100–400 mm with the interval of 100 mm, respectively. In the following experiments, the reference lengths on the scaleplate are 30–120 mm with the interval of 30 mm. When the scaleplate-projector distance is 100 mm, the recovered errors of the benchmark lengths are displayed in Fig. 4a. The error absolute means obtained are 0.473 mm, 0.550 mm, 0.593 mm and 0.931 mm, respectively. In Fig. 4b, the scaleplate-projector distance is 200 mm. The means of error absolutes are 0.589 mm, 0.626 mm, 0.678 mm and 1.086 mm, respectively. For the scaleplate-projector distance of 300 mm in Fig. 4c, the error absolute averages obtained by the invariant-based method are 0.719 mm, 0.840 mm, 1.178 mm and 1.394 mm, respectively. For the scaleplate-projector distance of 400 mm in Fig. 4d, the error absolute means obtained are 0.811 mm, 0.920 mm, 1.224 mm and 1.648 mm, respectively. In addition, the reference-camera distance grows up to 600 mm, the error absolute averages of the invariant reconstruction test are summarized in Fig. 5a. It can be concluded that the reconstruction error increases evidently when the scaleplate-projector distance rises from 100 to 400 mm. For a fixed scaleplate-projector distance, there construction error shows an obvious jump with the increasing reference length. Therefore, the error value is the smallest for the 600 mm reference-camera distance, the 100 mm scaleplate-projector distance, and the 30 mm reference length.

The reference-camera distance is determined by 700 mm secondly. The error absolutes of the invariant-based recovery are presented in Fig. 4e–h, and the averages of the errors are summarized in Fig. 5b. When the scaleplate-projector distance grows up from 100 to 400 mm, the error absolute averages are 0.923 mm, 1.147 mm, 1.549 mm and 1.842 mm that corresponds to the errors of 0.637 mm, 0.745 mm, 1.033 mm, 1.151 mm in above experiments, respectively. It shows that the reconstruction error grows up if the camera-reference distance increases from 600 to 700 mm.

The reference-camera distance is adjusted to 800 mm thirdly. The errors of the invariant-based method are shown in Fig. 4i–l. The error averages are shown in Fig. 5c. When the scaleplate-projector distance are 100–400 mm with the interval of 100 mm, the means of error absolutes increase to 1.980 mm, 2.540 mm, 2.702 mm and 3.090 mm, which are greater than the second group of experiments. It is evident from the observation of Fig. 5c that the reconstruction error grows while the scaleplate-laser distance climbs from 100 to 400 mm and the reference length increases from 30 to 120 mm.

The last test is carried out with a fixed reference-camera distance of 900 mm. The error absolutes and error averages are shown in Figs. 4m–p and 5d, separately. When the scaleplate-projector distance grows up from 100 to 400 mm, the error averages are 3.370 mm, 3.609 mm, 3.868 mm and 4.220 mm, respectively. The errors of the third group are lower than that of the last group, evidently. It can be globally concluded that the error achieves the smallest if the reference-camera distance is 600 mm, the scaleplate-projector distance is 100 mm, and the reference length is 30 mm.

## Summary

A vision-based approach to recover the 3D laser feature points is addressed and realized by the invariant composed of two points and the circle on 2D reference. First, the laser plane is regulated to be in the same plane of the 2D reference in this method. The position of the measured object can be observed flexibly by moving the 2D reference. The invariant is established by the circle, the reference point, the laser point in CSR and also by the projective cubic curve, the projective reference point and the projective laser point in CSI. Then, the reconstruction model of the laser point is constructed by the invariant characteristics and converted to CSC. Finally, the performance and reconstruction error of the method are verified by experiments. The minimum error is 0.473 mm for the camera-reference distance of 600 mm, the scaleplate-projector distance of 100 mm and the reference length of 30 mm. Besides, the maximum error is 4.960 mm, for the camera-reference distance of 900 mm, the scaleplate-projector distance of 400 mm and the reference length of 120 mm. The reconstruction error means are 0.891 mm, 1.365 mm, 2.578 mm and 3.767 mm with respect to the test conditions of 600–900 mm. The experiments prove that the point-circle invariant method is a promising and convenient measurement in the studies of shape reconstruction. The laser plane is positioned to the same plane of the 2D reference in the method. In the future research, the point-circle invariant method without the coplanarity constraint of the laser plane and reference will be investigated for wide applications.

## Data Availability

The datasets generated during the current study are available from the corresponding author on reasonable request.
